# Autonomous Multi-Robot Search for a Hazardous Source in a Turbulent Environment

**DOI:** 10.3390/s17040918

**Published:** 2017-04-21

**Authors:** Branko Ristic, Daniel Angley, Bill Moran, Jennifer L. Palmer

**Affiliations:** 1 School of Engineering, RMIT University, Melbourne, VIC 3000, Australia; daniel.angley@rmit.edu.au (D.A.); bill.moran@rmit.edu.au (B.M.); 2 Aerospace Division, Defence Science and Technology, Fishermans Bend, VIC 3207, Australia; jennifer.palmer@dsto.defence.gov.au

**Keywords:** cognitive search, biochemical source localisation, mobile robots

## Abstract

Finding the source of an accidental or deliberate release of a toxic substance into the atmosphere is of great importance for national security. The paper presents a search algorithm for turbulent environments which falls into the class of cognitive (*infotaxi*) algorithms. Bayesian estimation of the source parameter vector is carried out using the Rao–Blackwell dimension-reduction method, while the robots are controlled autonomously to move in a scalable formation. Estimation and control are carried out in a centralised replicated fusion architecture assuming all-to-all communication. The paper presents a comprehensive numerical analysis of the proposed algorithm, including the search-time and displacement statistics.

## 1. Introduction

Search strategies for finding an emitting source of hazardous substance based on sporadic sensory cues are a topic of great importance. In the context of national security, for example, the search could be for the source of an accidental or deliberate biochemical agent release into the atmosphere [1], which would require urgent attention. Similar techniques are also used in search and rescue missions and to explain the foraging behaviour of animals. In many studies of these search techniques, the searching agent is assumed to be mobile and capable of sensing the emitted substance from the source. The sensing cues are often sporadic, fluctuating and discontinuous, due to turbulent transport through the medium over a large search domain [2]. The objective of the search is to find the emitting source in the shortest possible time.

Recent advances in biochemical sensing technologies [3,4] have made the deployment of robots to search for such emitting sources very attractive [5]. Developing autonomous robots equipped with appropriate sensing capability to explore and search in harsh and dangerous environments, such as toxic or flammable ones, is the ultimate goal of this research. Due to its importance, the topic has attracted a great deal of interest from the scientific community. A survey including a taxonomy of pre-2008 search algorithms can be found in [6]. The dominant class of methods are bio-inspired strategies [7,8,9], which mimic the use of olfactory senses by bacteria, insects and other animals to localise food sources, detect predators, or find mates. Typically, the search actions depend on sensory cues. For example, upon sensing an odor signal, male moths surge upwind in the direction of the flow. When odor information vanishes, they exhibit random cross-wind casting or zigzagging to perform a local search until the plume is reacquired. Chemotaxis algorithms are a bio-inspired class of algorithms, in which the search is carried out by climbing the concentration gradient [10]. These strategies are effective close to the source where the odor plume can be considered as a continuous cloud. In the absence of positive detections, the robot may stay in one position, move linearly, or carry out a random walk. Once the chemical is detected, motion is directed towards higher gradients of concentration. These bio-inspired techniques are simple, as they require only limited spatial perception [11], however they are mostly ad hoc. Another popular class of algorithms, not mentioned in [6], are stochastic source-seeking algorithms [12,13,14]. While these algorithms are theoretically sound, they rely on smooth gradients of concentration. In the presence of turbulence, however, where the plume of the tracer randomly breaks up into time-varying disconnected patches resulting in intermittent measurements, these stochastic source-seeking algorithms are not practical.

Recently, a class of algorithms referred to as *infotaxis* [15] was developed specifically for searching in turbulent flows. In the absence of a smooth distribution of concentration, caused by turbulence, this strategy directs the robot towards the highest information gain. As a theoretically principled approach, with the source-term estimation being carried out in the Bayesian framework and the robot motion control being based on information-theoretic principles, the infotaxic strategy has been adopted by a number of research groups [16,17,18,19,20,21,22,23,24].

The goal of Bayesian estimation is to construct the posterior probability density function (PDF) of the source parameter vector, which typically includes its location, release rate and size. In [15] the estimation was carried out using an approximate grid-based nonlinear filtering technique [25] on a two-dimensional parameter space consisting of the *x* and *y* Cartesian coordinates of the source location. The source-release rate was assumed known, as well as the environmental parameters. The search was carried out using a single robotic platform. Grid-based nonlinear filtering was subsequently replaced with the sequential Monte Carlo method (also known as the particle filter) in [21,22,23,26]. The particle filter is more efficient than grid-based methods and is therefore capable of estimating the source-release rate jointly with the source location. Multi-robot infotaxis were investigated in [16,22,24].

Sequential estimation of the source posterior PDF using the particle filter is problematic when the state (the source parameter vector) is random, but *stationary* [27]. The absence of dynamics in the state implies that the exploration of the parameter space is limited to the first time index only. Even by artificially introducing dynamics to the state (e.g. a random walk-like dynamic with a small variance), the depletion of particles is inevitable over time and causes the collapse of the PF for long-duration searches [23]. In this paper we apply a Rao–Blackwell dimension reduction method to estimate the posterior PDF of the source parameter vector. This method computes the source release-rate, conditioned on the source location, analytically and uses the Monte Carlo method only in the space of *x* and *y* coordinates of the source. As a batch method, it does not suffer from depletion of particles over time. This paper studies the multi-robot search assuming a replicated centralised fusion with all-to-all communication. The robots move in a scalable formation, where the maximum size of the formation is determined by the specified communication range. Control actions are made autonomously by all robots using entropy reduction as the information theoretic measure. In computing the reward, however, we also introduce a cost of motion. The paper analyses the statistical patterns of the search, in particular the formation displacements and its scale.

The organisation of the paper is as follows. Mathematical models are presented in Section 2. The method of source parameter estimation is described in Section 3. Robotic platform motion control is explained in Section 4. Section 5 presents the numerical results, through simulations and using an experimental dataset characterised by a turbulent flow. Finally, the conclusions are drawn in Section 6.

## 2. Mathematical Models

### 2.1. Robot Motion Model

In this section we describe the motion model of each individual robotic platform. Suppose there are N≥1 robotic platforms, with the pose of the *i*th platform at time $t_{k}$ denoted by a vector $\theta_{k}^{i}={\left[{\left(r_{k}^{i}\right)}^{⊺},\;\phi_{k}^{i}\right]}^{⊺}$, i=1,⋯,N. Here $r_{k}^{i}={\left(x_{k}^{i},y_{k}^{i}\right)}^{⊺}$ is the robot location and $\phi_{k}^{i}$ is its heading. The motion of the group of robots is coordinated, i.e they move in a formation. In particular, we use a circular formation with the *N* robots spread approximately equally on the perimeter of a circle (the formation is controlled to be equally spread on a circle, but due to process noise in the motion model, this can be only approximately achieved), whose center at time $t_{k}$ is $\left(x_{k}^{c},y_{k}^{c}\right)$ with the radius $\rho_{k}\in \left[r_{\min},r_{\max}\right]$. The minimum radius $r_{\min}$ is introduced to prevent the robots from colliding; the maximum radius $r_{\max}$ is to ensure all-to-all communication amongst the platforms. The bearing of the *i*th platform within the formation is fixed, while its radius is allowed to vary. The position of the *i*th platform is defined by the offset $\left(\Delta x_{i},\Delta y_{i}\right)$ from the centre $\left(x_{k}^{c},y_{k}^{c}\right)$. All platforms in the formation have approximately equal orientation, while the offset is given by:

(1) $$\Delta x_{i}=2\rho \cos\left(2\pi i/N\right),\;\Delta y_{i}=2\rho \sin\left(2\pi i/N\right)$$

for i=1,⋯,N.

**Figure 1:**
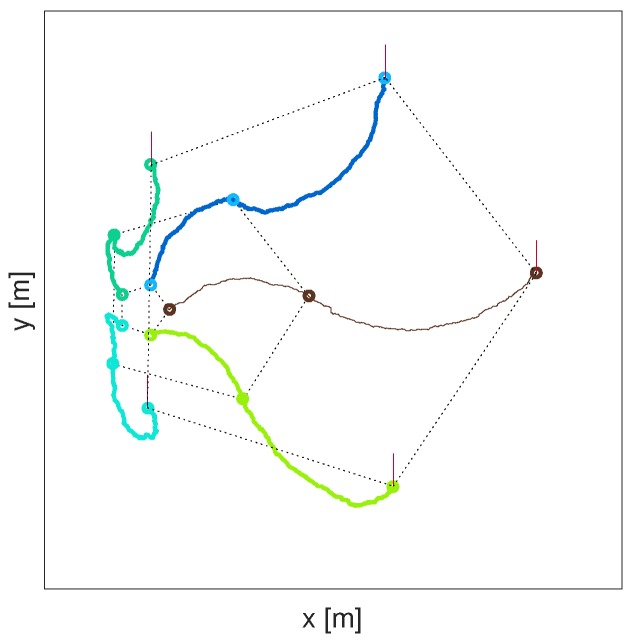
An illustration of a path of a coordinated group of N=5 searching robots at three consecutive time instants, with the scale of the formation increasing. The small circles in the figure represent robot locations $r_{k}^{i}$; the vertical lines indicate the current headings $\phi_{k}^{i}$, for i=1,⋯,N.

The biochemical sensors are activated at time instants $t_{k}$, k=1,2,⋯ to report concentration measurements. During the interval of time $\left[t_{k-1},t_{k}\right]$ between two consecutive sensing instants, the formation is moving. The duration of this interval, $T_{k}=t_{k}-t_{k-1}\geq 0$, is referred to as the *travel time*. The assumption is that sensing is suppressed during the travel time. The motion of the formation during the interval $\left[t_{k-1},t_{k}\right]$ is controlled by the following input parameters: the linear velocity $V_{k}$, the angular velocity $\Omega_{k}$, and the formation scale increment $\Delta \xi_{k}=\xi_{k}-\xi_{k-1}$ (which can be positive or negative). Given the control vector $u_{k}={\left[V_{k},\Omega_{k},\xi_{k},T_{k}\right]}^{⊺}$, the dynamics of *i*th platform during a short integration time [t−δ,t], where $t\in [t_{k-1}+\delta ,t_{k}]$ and $\delta \ll T_{k}$, can be modelled by a Markov process with the transitional density $\pi \left(\theta_{t}^{i}|\theta_{t-\delta }^{i},u_{k}\right)=N\left(\theta_{t}^{i};\beta \left(\theta_{t-\delta }^{i},u_{k}\right),\;Q_{k}\right)$. The mean of this normal density, $\beta (\theta_{t-\delta }^{i},u_{k})$, is a nonlinear function specified as follows:

(2) $$\beta \left(\theta_{t-\delta }^{i},u_{k}\right)=\theta_{t-\delta }^{i}+\delta \left[\begin{array}{c} V_{k}\;\cos\left(\phi_{k-1}^{i}\right)\\ V_{k}\;\sin\left(\phi_{k-1}^{i}\right)\\ \Omega_{k} \end{array}\right]+B+C$$

where:

$$B=\frac{\left(t-t_{k-1}\right)\Delta \xi_{k}}{T_{k}}\left[\begin{array}{c} \Delta x_{i}\\ \Delta y_{i}\\ 0 \end{array}\right],\;t\in \left[t_{k-1}+\delta ,t_{k}\right],$$

describes the motion due to the formation-scale change. Vector *C* in (2) introduces the corrections to the pose in order to remove the difference between the desired (deterministic) pose:

$${\bar{\theta }}_{k-1}^{i}=\frac{1}{N}\sum_{j=1}^{N}\theta_{k-1}^{j}+\xi_{k-1}\left[\begin{array}{c} \Delta x_{i}\\ \Delta y_{i}\\ 0 \end{array}\right]$$

and the actual pose (perturbed by process noise) $\theta_{k-1}^{i}$ at the previous sensing time $t_{k-1}$, i.e., $C=-\left({\bar{\theta }}_{k-1}^{i}-\theta_{k-1}^{i}\right)\left(t_{k}-t\right)/T_{k}$. Without the correction term *C*, the platforms could drift and break the formation during the search time. Finally, $Q_{k}$ is the covariance matrix which accounts for stochastic disturbances in motion. Figure 1 illustrates a simulated path of a robot formation based on the described motion model. In this illustration, the formation of N=5 robots is expanding (the scales are $\xi_{1}=1$, $\xi_{2}=4$ and $\xi_{3}=8$). Note that, despite random individual paths, the formation at measurement time instants $t_{1},t_{2},t_{3}$ is almost a perfect pentagon. Apart from some randomness due to process noise, the motion model keeps the formation with the fixed bearings relative to its centre and a fixed north (i.e., the formation does not rotate).

### 2.2. Measurement Model

Dispersion of the emitted hazardous substance in a turbulent environment is modelled using the Lagrange particle encounters model developed in [15]. Suppose that the emitting source is located at coordinates $r_{0}={\left(X_{0},Y_{0}\right)}^{⊺}$ and its release rate, or strength, is $Q_{0}$. The source parameter vector is denoted $\eta ={\left[r_{0}^{⊺}\;Q_{0}\right]}^{⊺}$. The particles released from the source propagate with combined molecular and turbulent isotropic diffusivity *D*, but can also be advected by wind. The released particles have an average lifetime of τ before being absorbed. Let the *average* wind characteristics be the speed *U* and direction, which by convention, coincides with the direction of the *x* axis.

Suppose a spherical sensor of small radius *a* is mounted on the *i*th robot platform, whose pose at time *k* is $\theta_{k}^{i}={\left[x_{k}^{i},\;y_{k}^{i},\;\phi_{k}^{i}\right]}^{⊺}$ (robot location is assumed non-coincidental with the source location $r_{0}$). This sensor will experience a series of encounters with the particles released from the emitting source. The average rate of encounters can be modelled as follows [15]:

(3) $$R_{\eta }\left(r_{k}^{i}\right)=\frac{Q_{0}}{\ln\left(\frac{\lambda }{a}\right)}\;\exp\left[\frac{(X_{0}-x_{k}^{i})U}{2D}\right]\cdot K_{0}\left(\frac{d_{k}^{i}\left(r_{0}\right)}{\lambda }\right)$$

where *D*, τ and *U* are known environmental parameters,

(4) $$d_{k}^{i}\left(r_{0}\right)=\sqrt{{\left(x_{k}^{i}-X_{0}\right)}^{2}+{\left(y_{k}^{i}-Y_{0}\right)}^{2}}$$

is the distance between the source and *i*th sensor platform, $K_{0}$ is the modified Bessel function of the second kind of order zero, and

(5) $$\lambda =\sqrt{\frac{D\tau }{1+\frac{U^{2}\tau }{4D}}}.$$

depends on environmental parameters only.

The stochastic process of sensor encounters with the dispersed particles is modelled by a Poisson distribution. The probability that a sensor at location $r_{k}^{i}$ encounters $z\in Z^{+}\cup \left\{0\right\}$ particles (*z* is a non-negative integer) during a time interval $t_{0}$ is then:

(6) $$P\left(z;\mu_{k}^{i}\right)=\frac{{\left(\mu_{k}^{i}\right)}^{z}}{z!}e^{-\mu_{k}^{i}}$$

where $\mu_{k}^{i}=t_{0}\cdot R_{\eta }\left(r_{k}^{i}\right)$ is the mean number of particles expected to reach the sensor at location $r_{k}^{i}$ during $t_{0}$.

## 3. Source Parameter Estimation

### 3.1. Problem Specification

Let $z_{k}^{i}$ denote the sensor measurement recorded by the *i*th robot platform at time $t_{k}$. The sequence of such measurements from platform *i*, starting from the beginning of the search until $t_{k}$ is a vector $z_{1:k}^{i}={\left[z_{1}^{i},\cdots ,z_{k}^{i}\right]}^{⊺}$, i=1,⋯,N. Similarly, the measurements from all platforms up to time $t_{k}$ is a vector $z_{1:k}={\left[{\left(z_{1:k}^{1}\right)}^{⊺},\cdots ,{\left(z_{1:k}^{N}\right)}^{⊺}\right]}^{⊺}$. Accordingly we can define the vector of sensor measurement locations corresponding to $z_{1:k}$ as $r_{1:k}={\left[r_{1}^{1},\cdots ,r_{k}^{1},\cdots ,r_{1}^{N},\cdots ,r_{k}^{N}\right]}^{⊺}$. This vector is assumed to be known. Whenever in the text we refer to the measurement vector $z_{1:k}$, we implicitly assume that it is in pair with $r_{1:k}$.

Assuming all-to-all communication between the platforms for exchanging mutually their latest measurements and positions (ignoring the time-delays and bandwidth limitations), $z_{1:k}$ and $r_{1:k}$ are available at every platform at time $t_{k}$ for processing. Thus, every platform can independently carry out centralised fusion in order to estimate the source parameter vector η. This type of fusion architecture is known as replicated centralised.

Assuming the sensor measurements, conditioned on η, are independent, the likelihood function of the measurement vector $z_{1:k}$ can be written as a double product $\ell \left(z_{1:k}|\eta \right)=\prod_{i=1}^{N}\prod_{j=1}^{k}P\left(z_{j}^{i};t_{0}R_{\eta }\left(r_{j}^{i}\right)\right)$.

We adopt the Bayesian estimation framework with the goal of computing the posterior PDF $p(\eta |z_{1:k})$. In this framework, in addition to $\ell (z_{1:k}|\eta )$, one needs to specify the prior distribution of the parameter vector π(η). Then, using the Bayes’ rule, the posterior PDF is:

(7) $$p\left(\eta |z_{1:k}\right)=\frac{\ell \left(z_{1:k}|\eta \right)\pi \left(\eta \right)}{\int \ell \left(z_{1:k}|\eta \right)\pi \left(\eta \right)d\theta }.$$

For the described problem, (7) cannot be solved analytically and we need to apply a numeric approximation. However, it will be shown that, assuming the prior of the source strength $\pi (Q_{0})$ is a gamma distribution, the posterior $p(Q_{0}|z_{1:k},r_{0})$ can be solved analytically. Because the posterior $p(\eta |z_{1:k})$, using the chain rule, can be expressed as:

(8) $$p\left(\eta |z_{1:k}\right)=p\left(Q_{0}|z_{1:k},r_{0}\right)\;p\left(r_{0}|z_{1:k}\right),$$

x we will only need to apply a numeric approximation to estimate the posterior $p(r_{0}|z_{1:k})$.

### 3.2. Solution

It is reasonable to assume that the source strength is independent of its location, and hence $\pi \left(\eta \right)=\pi \left(Q_{0}\right)\pi \left(r_{0}\right)$. Let us adopt for $\pi (Q_{0})$ a gamma distribution, with the shape parameter $\kappa_{0}$ and the scale parameter $\vartheta_{0}$:

(9) $$\pi \left(Q_{0}\right)=G\left(Q_{0};\kappa_{0},\vartheta_{0}\right)=\frac{Q_{0}^{\left(\kappa_{0}-1\right)}\;e^{-Q_{0}/\vartheta_{0}}}{\vartheta_{0}^{\kappa_{0}}\;\Gamma \left(\kappa_{0}\right)}.$$

Note that for suitably chosen hyperparameters $\kappa_{0}$ and $\vartheta_{0}$, the support of this prior can cover a large span of possible values of $Q_{0}$.

The conjugate prior of the Poisson distribution is the gamma distribution [28]. Therefore, the posterior $p(Q_{0}|r_{0},z_{1:k})$ is also a gamma distribution, $p\left(Q_{0}|r_{0},z_{1:k}\right)=G\left(Q_{0};\kappa_{k},\vartheta_{k}\right)$, with parameters $\kappa_{k}$ and $\vartheta_{k}$ expressed analytically as a function of $r_{0}$ and $z_{1:k}$ as follows (see Appendix A):

(10) $$\begin{array}{ccc} \kappa_{k} & = & \kappa_{0}+\sum_{i=1}^{N}\sum_{j=1}^{k}z_{j}^{i}, \end{array}$$

(11) $$\begin{array}{ccc} \vartheta_{k} & = & \frac{\vartheta_{0}}{1+\vartheta_{0}\sum_{i=1}^{N}\sum_{j=1}^{k}\rho_{r_{0}}\left(r_{j}^{i}\right)}. \end{array}$$

where $\rho_{r_{0}}\left(r_{k}^{i}\right)=t_{0}\;R_{\eta }\left(r_{k}^{i}\right)/Q_{0}$, that is:

(12) $$\rho_{r_{0}}\left(r_{k}^{i}\right)=\frac{t_{0}}{\ln\left(\frac{\lambda }{a}\right)}\;\exp\left[\frac{(X_{0}-x_{k}^{i})U}{2D}\right]\cdot K_{0}\left(\frac{d_{k}^{i}\left(r_{0}\right)}{\lambda }\right).$$

It remains to compute the posterior PDF $p(r_{0}|z_{1:k})$:

(13) $$p\left(r_{0}|z_{1:k}\right)=\frac{g\left(z_{1:k}|r_{0}\right)\pi \left(r_{0}\right)}{\int g\left(z_{1:k}|r_{0}\right)\pi \left(r_{0}\right)dr_{0}}.$$

Compared to (7), which is a three-dimensional PDF, the posterior in (13) is two-dimensional. This dimension reduction improves the accuracy of the numerical solution. After a few lines of mathematical derivations one can show that the analytic expression for the likelihood function $g(z_{1:k}|r_{0})$, which features in (13), is given by (see Appendix A):

(14) $$\begin{array}{cc} g(z_{1:k}|r_{0})= & \vartheta_{0}^{\sum_{i=1}^{N}\sum_{j=1}^{k}z_{j}^{i}}\;\frac{\Gamma \left(\kappa_{0}+\sum_{i=1}^{N}\sum_{j=1}^{k}z_{j}^{i}\right)}{\Gamma (\eta_{0})}\cdot \prod_{i=1}^{N}\prod_{j=1}^{k}\frac{{\left[\rho_{r_{0}}\left(r_{j}^{i}\right)\right]}^{z_{j}^{i}}}{z_{j}^{i}!}. \end{array}$$

A plethora of techniques is available for the numerical computation of (13), from grid-based numerical integration methods to Monte Carlo methods (e.g. the Markov Chain Monte Carlo, importance sampling and population Monte Carlo). We choose a Monte Carlo method which, for a given $\pi (r_{0})$ and $z_{1:k}$, approximates the posterior PDF $p(r_{0}|z_{1:k})$ by a random sample ${\left\{r_{0,k}^{\left(m\right)}\right\}}_{1\leq m\leq M}$ as follows:

$$p\left(r_{0}|z_{1:k}\right)\approx p_{M}\left(r_{0}|z_{1:k}\right)=\frac{1}{M}\sum_{m=1}^{M}\delta \left(r_{0}-r_{0,k}^{\left(m\right)}\right).$$

Here $\delta (r_{0}-r_{0,k}^{\left(m\right)})$ denotes the delta-Dirac mass located at $r_{0,k}^{\left(m\right)}$. As the size of the sample M→∞, the moments of $p_{M}\left(r_{0}|z_{1:k}\right)$ converge almost surely to the moments of $p(r_{0}|z_{1:k})$. An advantage of the Monte Carlo method over the grid-based (deterministic) integration is that the former positions its integration points (i.e., samples) in the regions of high probability [29].

The basic steps in estimation of $p(\eta |z_{1:k})$ expressed by (8) are summarised in Algorithm 1. The expected *a posteriori* point estimates of the source location and its strength can be computed from the output ${\left\{\left(r_{0,k}^{\left(m\right)},\kappa_{k},\vartheta_{k}^{\left(m\right)}\right)\right\}}_{1\leq m\leq M}$ as follows:

(15) $$\begin{array}{ccc} {\hat{r}}_{0} & = & \frac{1}{M}\sum_{m=1}^{M}r_{0,k}^{\left(m\right)} \end{array}$$

(16) $$\begin{array}{ccc} {\hat{Q}}_{0} & = & \frac{1}{M}\sum_{m=1}^{M}\kappa_{k}\cdot \vartheta_{k}^{\left(m\right)} \end{array}$$

**Algorithm 1** Estimation of $p(\eta |z_{1:k})$1:Input: $\pi (r_{0})$, $\kappa_{0}$, $\vartheta_{0}$, $z_{1:k}$, *M*
2:Estimate $p(r_{0}|z_{1:k})$ by a random sample ${\left\{r_{0,k}^{\left(m\right)}\right\}}_{1\leq m\leq M}$
3:Compute $\kappa_{k}$, Equation (10) 4:Compute $\vartheta_{k}^{\left(m\right)}$ using $r_{0,k}^{\left(m\right)}$, Equation (11); m=1,⋯,M
5:Output: ${\left\{\left(r_{0,k}^{\left(m\right)},\kappa_{k},\vartheta_{k}^{\left(m\right)}\right)\right\}}_{1\leq m\leq M}$


Finally, the Monte Carlo method which estimates $p(r_{0}|z_{1:k})$ in line 2 of Algorithm 1, was implemented using iterated importance sampling with progressive correction (IIS-PC) [30]. Full details of the implementation are given in [31]. A desirable property of importance sampling is to draw samples from an importance density that result in sample weights with a minimal variance. The question is how to design this importance density. The key idea behind IIS-PC is to achieve the goal of drawing samples with a minimal variance in a sequential manner, by constructing a sequence of target distributions from which to draw samples. The first target distribution is the prior, while every subsequent target distribution should increasingly resemble the posterior. A target distribution which can be used in this context at iteration s=1,⋯,H of the IIS-PC is:

(17) $$p_{s}\left(r_{0}|z_{1:k}\right)\propto {\left[g(z_{1:k}|r_{0})\right]}^{\Gamma_{s}}\pi \left(r_{0}\right),$$

where $g(z_{1:k}|r_{0})$ is given by (14), $\Gamma_{s}=\sum_{v=1}^{s}\gamma_{v}$ with $\gamma_{v}\in \left(0,1\right]$ and $\Gamma_{H}=1$. Note that $\Gamma_{s}$ is an increasing function of *s*, upper bounded by one. As a consequence, the intermediate likelihood is broader than the true likelihood, particularly in the early stages (for small *s*). Thus, the sequence of target distributions in (17) gradually introduces the correction imposed by the measurement $z_{k}$ on the prior $\pi (r_{0})$. To derive any benefits from IIS-PC, it is required after each stage to remove the lowly weighted members of the sample and diversify the remaining ones. Lowly weighted members are removed by resampling [27], while diversification of the remaining samples is performed by Markov transitions whose stationary distribution is the target distribution $p_{s}\left(r_{0}|z_{1:k}\right)$. The outcome is a diverse sample located in the region of the parameter space where the intermediate likelihood has non-zero values. The choice of correction factors $\gamma_{1},\cdots ,\gamma_{H}$, as well as the number of iterations *H* are design parameters. Further details can be found in [31].

## 4. Robot Formation Control

Suppose at time $t_{k-1}$ the robotic platforms have processed all available measurements included in the vector $z_{1:k-1}$ and estimated the posterior PDF $p(\eta |z_{1:k-1})$ using the method described in Section 3. Let $A\subset R^{2}$ denote a designated search area, which includes the source location $r_{0}$. The key aspect of search is, for each robotic platform of the formation, to decide autonomously where to move next within A in order to acquire a new concentration measurement at $t_{k}$, denoted $z_{k}={\left[z_{k}^{1},\;z_{k}^{2},\cdots ,z_{k}^{N}\right]}^{⊺}$.

An autonomous multi-robot search can be formulated as a partially-observed Markov decision process (POMDP) [32]. The elements of a POMDP include an information state, the space of admissible actions (controls) and a reward function. The information state, adopting the Bayesian framework for estimation of source parameters, is the posterior PDF $p(\eta |z_{1:k-1})$. Current knowledge about the source position and strength is fully specified by this density. A decision in the context of search is the selection of a control vector $u_{k}\in U_{k}$, where $U_{k}$ is the space of admissible actions. Finally, the reward function maps each admissible action into a non-negative real number, which represents a measure of the action’s expected information gain. An optimal strategy selects, at each time, the action with the highest reward. Admissible actions can be formed with one or multiple steps ahead. According to the motion model introduced in Section 2.1, the space of admissible actions $U_{k}$ is continuous with four dimensions: $V_{k}$, $\Omega_{k}$, $\xi_{k}$ and $T_{k}$. In order to reduce the computational complexity of the numerical optimisation, we discretise $U_{k}$ and consider only myopic (one step ahead) control.

If V, O, S and T denote the sets of possible discrete-values of $V_{k}$, $\Omega_{k}$, $\xi_{k}$ and $T_{k}$, respectively, then $U_{k}$ is the Cartesian product V×O×S×T. The myopic selection of the control vector at time $t_{k}$ is expressed as:

(18) $$u_{k}=\arg\max_{v\in U_{k}}E\left\{D\left(p\left(\eta |z_{1:k-1}\right),z_{k}\left(v\right)\right)\right\}$$

where $D\left(p\left(\eta |z_{1:k-1}\right),z_{k}\left(v\right)\right)$ is the reward function. Note that the reward function depends on the future measurement $z_{k}\left(v\right)$, assuming the control vector $v\in U_{k}$ has been applied. In reality, this future measurement is not available (the decision has to be made at time $t_{k-1}$), and therefore the expectation operator E with respect to the prior measurement PDF features in (18). Previous studies of search strategies [16,23] found that the reward function measuring the information gain as the *entropy reduction*, results in the most efficient search. However, the earlier work neglected that, while traveling, robots incur certain cost. Assuming α is the cost of travel per unit distance, we adopt the following specification for the expected reward function:

(19) $$\begin{array}{c} E\left\{D\left(p\left(\eta |z_{1:k-1}\right),z_{k}\left(v\right)\right)\right\}=\\ \;\left[H_{k-1}-E\left\{H_{k}\left(z_{k}\left(v\right)\right)\right\}\right]\;e^{-\alpha \;s_{k}\left(v\right)} \end{array}$$

where $s_{k}\left(v\right)=V_{k}T_{k}\geq 0$ is the travelled distance under action v, $H_{k-1}$ is the current entropy (based on $z_{1:k-1}$), i.e.,

(20) $$H_{k-1}=-\int p\left(\eta |z_{1:k-1}\right)\;\ln p\left(\eta |z_{1:k-1}\right)d\eta ,$$

$H_{k}\left(z_{k}\left(v\right)\right)$ is the future entropy (after applying the control v):

(21) $$H_{k}\left(z_{k}\left(v\right)\right)=-\int p\left(\eta |z_{1:k-1},z_{k}\left(v\right)\right)\ln p\left(\eta |z_{1:k-1},z_{k}\left(v\right)\right)d\eta .$$

and $E\{H_{k}\left(z_{k}\left(v\right)\right)\}$ is its expected value, with respect to the probability mass function $P\left\{z_{k}|z_{1:k-1}\right\}=\int \ell \left(z_{k}|\eta \right)\;p\left(\eta |z_{1:k-1}\right)d\eta$:

(22) $$E\left\{H_{k}\left(z_{k}\left(v\right)\right)\right\}=\sum_{z_{k}}P\left\{z_{k}|z_{1:k-1}\right\}\cdot H_{k}\left(z_{k}\left(v\right)\right).$$

The exponential term, $e^{-\alpha \;s_{k}\left(v\right)}$, in (19) incorporates the cost of travel, penalising longer travel distances.

The computation of entropy $H_{k-1}$ in (20) is carried out as follows. Recall that $p(\eta |z_{1:k-1})$ is approximated by a random sample ${\left\{\left(r_{0,k-1}^{\left(m\right)},\kappa_{k-1},\vartheta_{k-1}^{\left(m\right)}\right)\right\}}_{1\leq m\leq M}$. From this representation, one can compute a random sample ${\left\{\eta_{k-1}^{\left(m\right)}\right\}}_{1\leq m\leq M}$, with uniform weights 1/M, where $\eta_{k-1}^{\left(m\right)}={\left[{\left(r_{0,k-1}^{\left(m\right)}\right)}^{⊺},\;Q_{0,k-1}^{\left(m\right)}\right]}^{⊺}$ and $Q_{0,k-1}^{\left(m\right)}∼G\left(Q_{0};\kappa_{k-1},\vartheta_{k-1}^{\left(m\right)}\right)$, for m=1,⋯,M. Then $p\left(\eta |z_{1:k-1}\right)\approx \frac{1}{M}\sum_{m=1}^{M}\delta \left(\eta -\eta_{k-1}^{\left(m\right)}\right)$, which leads to the approximation $H_{k-1}\approx -\frac{1}{M}\ln\frac{1}{M}$.

The computational expense of exact computation of $E\{H_{k}\left(z_{k}\left(v\right)\right)\}$ grows exponentially with *N* because the sum in (22) is *N*-dimensional: it requires consideration of all possible combinations of measurement outcomes from *N* platforms. Hence we resort to a Monte Carlo approximation. For a command vector $v\in U_{k}$, first we draw a random sample ${\left\{z_{k}^{\left(j\right)}\right\}}_{1\leq j\leq J}$ from $P\{z_{k}|z_{1:k-1}\}$ using the following procedure. We randomly select a sample $\eta_{k-1}^{*}$ from ${\left\{\eta_{k-1}^{\left(m\right)}\right\}}_{1\leq m\leq M}$, and then draw *N* times from the likelihood $\ell (z_{k}|\eta_{k-1}^{*})$. By repeating this procedure *S* times, J=SN samples of the measurement outcomes from *N* platforms ${\left\{z_{k}^{\left(j\right)}\right\}}_{1\leq j\leq J}$ are created. Then (22) is simply approximated as: $E\left\{H_{k}\left(z_{k}\left(v\right)\right)\right\}=\frac{1}{J}\sum_{j=1}^{J}H_{k}\left(z_{k}^{\left(j\right)}\left(v\right)\right)$.

The search algorithm needs to decide when to stop the search and report its final estimates of source parameters, denoted ${\hat{r}}_{0}$ and ${\hat{Q}}_{0}$. The termination criterion is based on the spatial spread of the random sample ${\left\{r_{0,k}^{\left(m\right)}\right\}}_{1\leq k\leq M}$, computed as the trace of its sample covariance matrix. When this spread is below a certain threshold, denoted ϖ, the search is terminated.

The key assumption of the replicated centralised fusion architecture is that the same input data (measurements $z_{1:k}$ and the corresponding locations $r_{1:k}$) are available to all robotic platforms for data fusion (parameter estimation and robot motion control). However, this assumption is not sufficient. As the Monte Carlo method plays a role in the data fusion, we must also ensure that the same pseudo-random generators, using the same seed, are running on each individual platform. In this way all platforms reach the same decision on the motion control vector $u_{k}$ and subsequently apply the motion model described in Section 2.1, knowing their own identification number *i* in the formation.

## 5. Numerical Results

### 5.1. Illustrative Run

First we illustrate a typical run of the multi-robot search algorithm using the following parameters (all physical quantities are in arbitrary units (a. u.)):

- True source parameters: $X_{0}=-150$, $Y_{0}=150$, $Q_{0}=4$;
- Search area A=500×500 and number of platforms N=5;
- Motion model parameters: $r_{\min}=1$, $r_{\max}=100$, δ=0.25, V={1}, O={−5,−2.5,0,2.5,5}, S={1,2,4,8}, T={0.25, 0.5,1,2,4,8,16,32,64,128,256}, and based on (1), the initial scale is $\xi_{0}=2$;
- Measurement model parameters: a=1, D=1, τ=250, U=0.25, $t_{0}=1$;
- Algorithm parameters: $\kappa_{0}=3$, $\vartheta_{0}=5.2$, M=1000, $\pi (r_{0})$ is the uniform distribution over the search area A, J=M, the cost of travel α=0.01, termination threshold ϖ=6.25.

**Figure 2:**
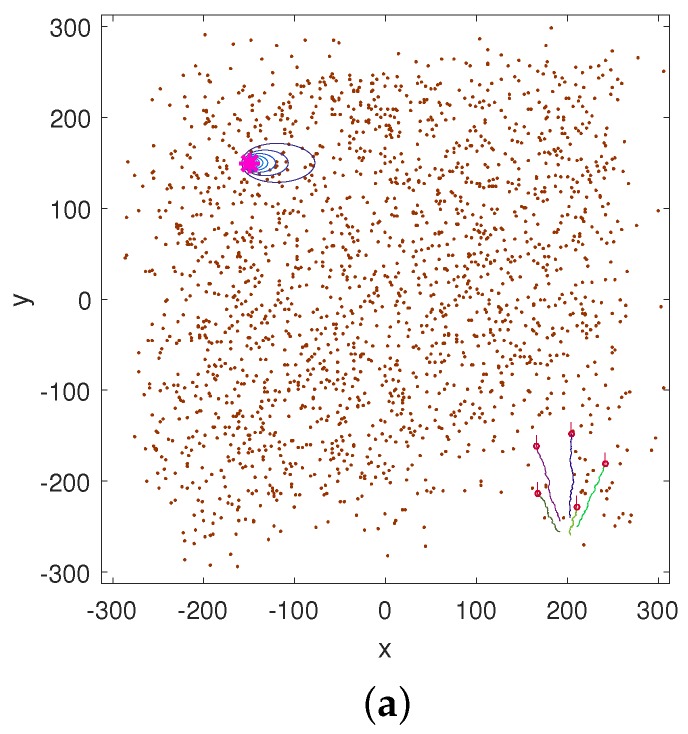

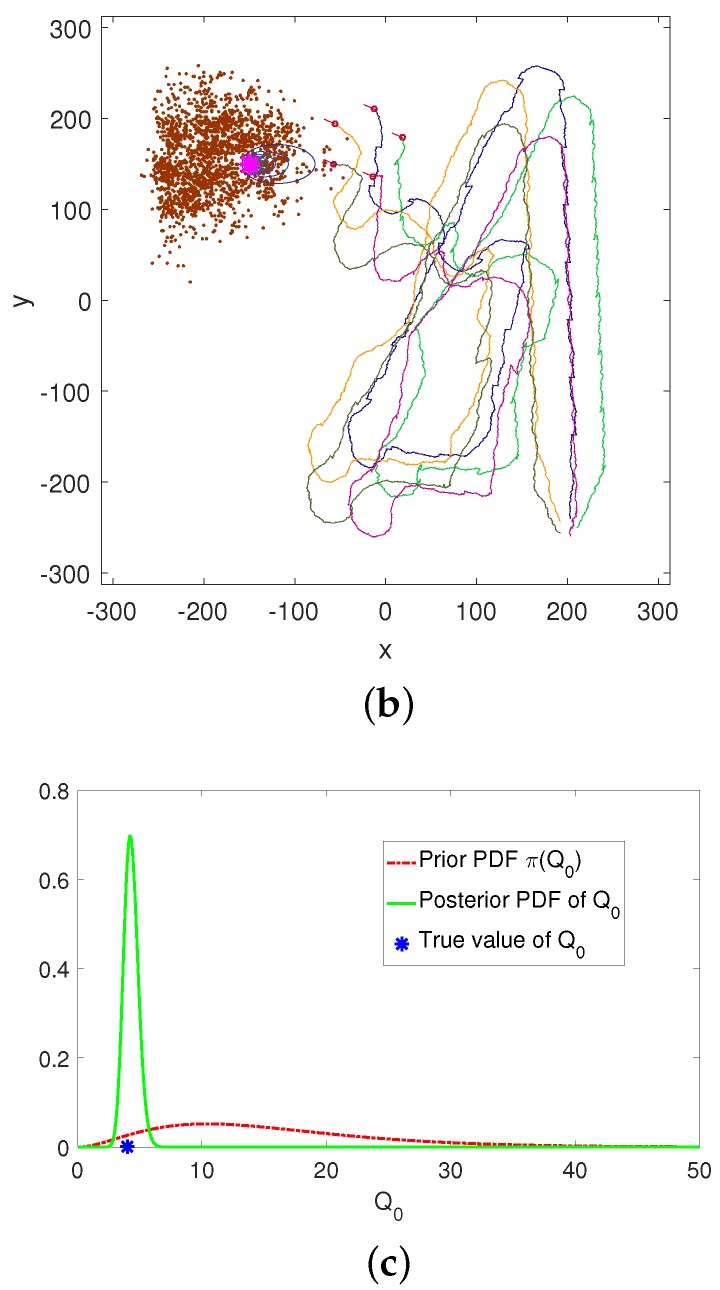
An illustrative run of the multi-robot search algorithm. Figures (**a**) and (**b**) show the search area, the paths of N=5 platforms, and the Monte Carlo samples (brown coloured dots) ${\left\{r_{0,k}^{\left(m\right)}\right\}}_{1\leq m\leq M}$ at k=2 and k=33, respectively. The true source location is indicated by a pink asterisk. The contours of the mean plume are plotted with blue lines. Figure (**c**) shows the prior probability density function (PDF) $\pi (Q_{0})$ (red dashed line), the posterior PDF $p(Q_{0}|r_{0},z_{1:k})$ at k=48 (green solid line), and the true value of $Q_{0}=4$ (blue asterisk).

The initial position of the centroid of the formation was at (200,−250). The results of a typical run are shown in Figure 2. Figure 2a shows the search area, the paths of the multi-robot formation at k=2 and the source location at $\left(X_{0},Y_{0}\right)$ with the contour plot of the corresponding *mean* rate $R_{\eta }$ of (3). The random samples ${\left\{r_{0,k}^{\left(m\right)}\right\}}_{1\leq k\leq M}$, approximating the posterior $p(r_{0}|z_{1:k})$, are shown as brown dots. Note that the search area is more than ten times bigger than the area where the source can be sensed. Consequently, the measurements $z_{1:k}$ are initially zero vectors for a long time; on this occasion for k=1,⋯,32. At k=33 the first non-zero measurement is recorded by one of the sensors, resulting in the posterior PDF $p(r_{0}|z_{1:k=33})$, approximated by the sample ${\left\{r_{0,k=33}^{\left(m\right)}\right\}}_{1\leq k\leq M}$ shown in Figure 2b. This figure also shows the search paths of the formation until the first detection. The search continues until k=48, when the termination criterion is reached. The source parameter estimates at this stage are ${\hat{r}}_{0}=\left(-151.45,149.98\right)$ and ${\hat{Q}}_{0}=4.3$. The posterior PDF of $Q_{0}$ at k=48 is shown in Figure 2c. Note the narrow distribution of this posterior compared to the prior $\pi (Q_{0})$. The total search time of this run was 2092 a.u.

### 5.2. Monte Carlo runs

In order to understand the performance characteristics of the search algorithm, 200 Monte Carlo runs were performed of various scenarios. Unless otherwise specified, the parameters used were the same as specified in Section 5.1, but with the bigger search area, A=750×750.

**Figure 3:**
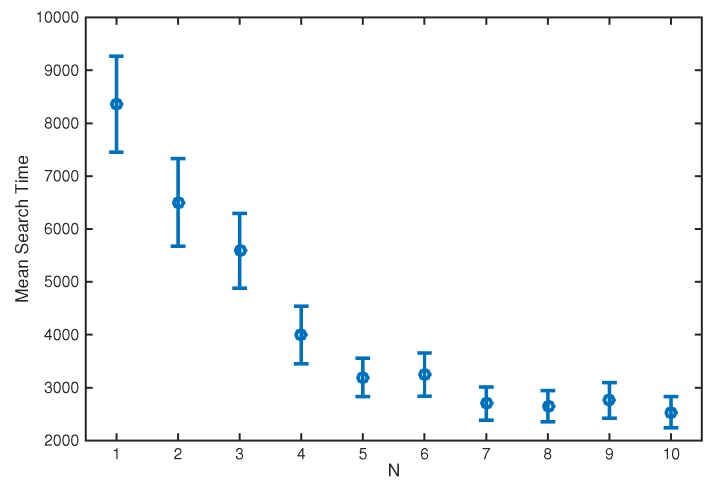
Mean search time when varying the number of platforms, *N*, from 1 to 10. The error bars show the 95% confidence interval for the estimate of the mean.

Figure 3 shows the mean search time when varying the number of platforms, *N*, from 1 to 10. Both the source location and initial centroid position were drawn at random from the uniform distribution over the search area. There is initially a significant decrease in the mean search time as platforms are added, but this eventually levels off. Even with a large number of platforms the formation still needs to get quite close to the source before there is a significant probability of a non-zero detection on any of the sensors, so the search time becomes dominated by the time spent exploring the area before that detection.

**Figure 4:**
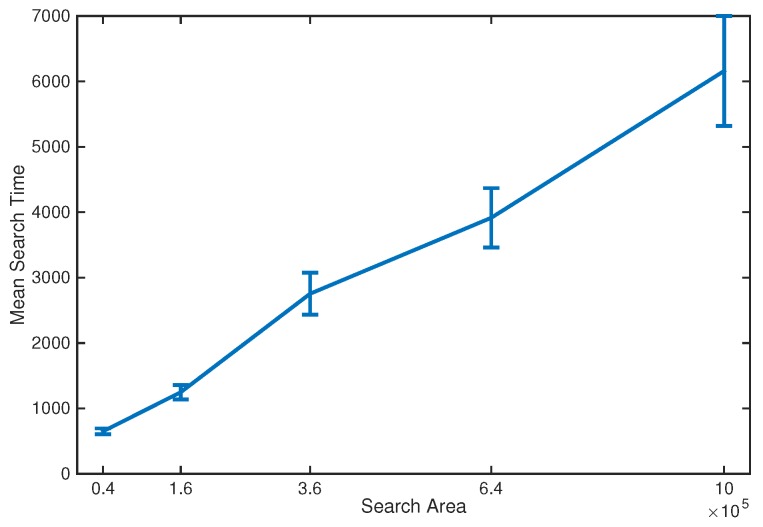
Mean search time for N=5 platforms when varying the side length of the search area from 200 to 1000. The error bars show the 95% confidence interval for the estimate of the mean.

Figure 4 shows the mean search time when varying the side length of the search area in the range [200,1000]. The search was performed using N=5 platforms and both the source location and initial centroid position were again drawn at random from the uniform distribution over the search area. Over this range of side lengths, the mean search time is approximately linear in the area searched.

**Figure 5:**
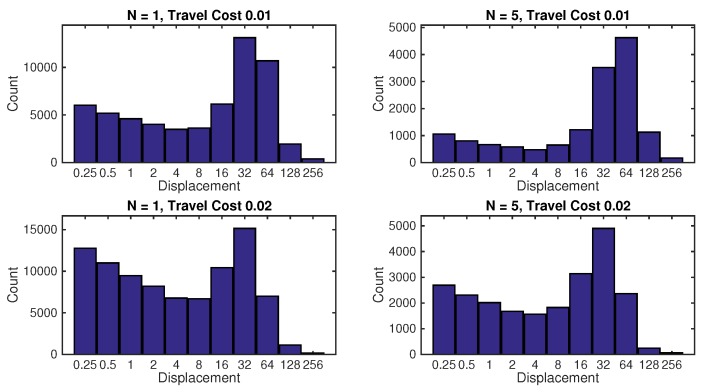
Histograms of robot formation displacements, as chosen by the search algorithm. The travel cost per unit distance was α=0.01 and α=0.02.

Figure 5 shows the effect of travel cost on the formation displacements chosen by the search algorithm. The travel cost α, was increased from 0.01 to 0.02 and the results are shown for 1 and 5 search platforms. In accordance with intuition, increasing the travel cost results in a shift of the histogram towards smaller displacements. Another interesting observation can be made from Figure 5: the histograms clearly has two peaks. One corresponds to the short movements, while the other peak corresponds to the long “jumps” of 32 or more units in length. This appears to be very similar to the displacement patterns of foraging animals [2,33,34]. They too typically combine the phases of long non-sensing displacement, with short sensing (and reactive) search phases. This strategy is often referred to as *intermittent search*.

**Figure 6:**
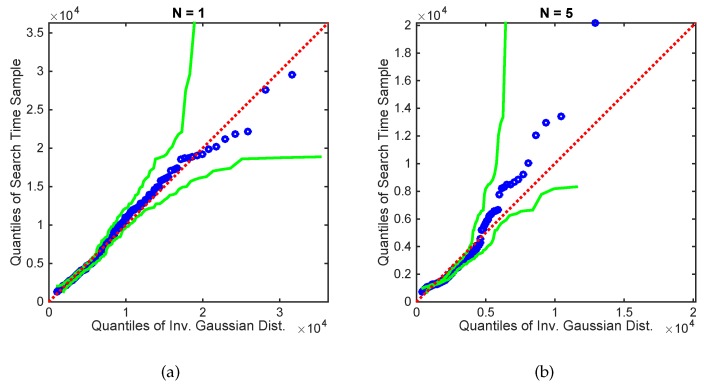
Q-Q plot of the search times for (**a**) N=1 and (**b**) N=5, versus the inverse Gaussian distribution with parameters fitted using maximum likelihood estimation. The source location was fixed at [187.5,187.5] and the initial centroid position at [187.5,−187.5]. The green lines show 95% confidence bands [35].

Figure 6 shows a Q-Q plot with 95% confidence intervals [35], comparing the empirical PDF of the search times (for N=1 and N=5 robotic platforms) with a fitted inverse Gaussian distribution. The match between the empirical and the proposed theoretical PDF can be accepted with 95% confidence, if the confidence limits, shown as green lines in Figure 6, do not cross the red dashed line. The empirical search time samples were obtained with the source location fixed at $\left(X_{0},Y_{0}\right)=\left(187.5,187.5\right)$ and the initial robot formation centroid at [187.5,−187.5]. As found in our previous work [23], the search time for a single search platform is well modelled by an inverse Gaussian. The model, however, does not hold as strongly for N=5 searching platforms, especially for shorter search times.

In all Monte Carlo runs of the proposed autonomous multi-robot search, the hazardous source was found and localised with accuracy determined by the termination criterion. In particular, the RMS localisation error was found to roughly correspond to $\sqrt{\varpi }=2.5$ a.u.

### 5.3. Experimental Results

The search algorithm was also evaluated on an experimental data set, collected by COANDA Research & Development Corporation using their large recirculating water channel. The source was releasing fluorescein dye at a constant rate from a narrow tube. The data is a sequence of 340 frames of instantaneous concentration field measurements in the vertical plane, sampled every 10/23 s. The size of each frame is 49×98 pixels, where each pixel corresponds to a square area of 2.935×2.935 mm${}^{2}$. The sequence of frames, in the form of a video, is included in the supplementary material; the actual dataset can be obtained from the authors on request.

The frames from this experimental dataset were scaled by a factor of 3 using bicubic scaling and placed in the top left corner of a 500×500 search area. The simulated measurements from the previous section were replaced with the rounded integer taken from the closest spatial and temporal sample from the experimental dataset.

**Figure 7:**
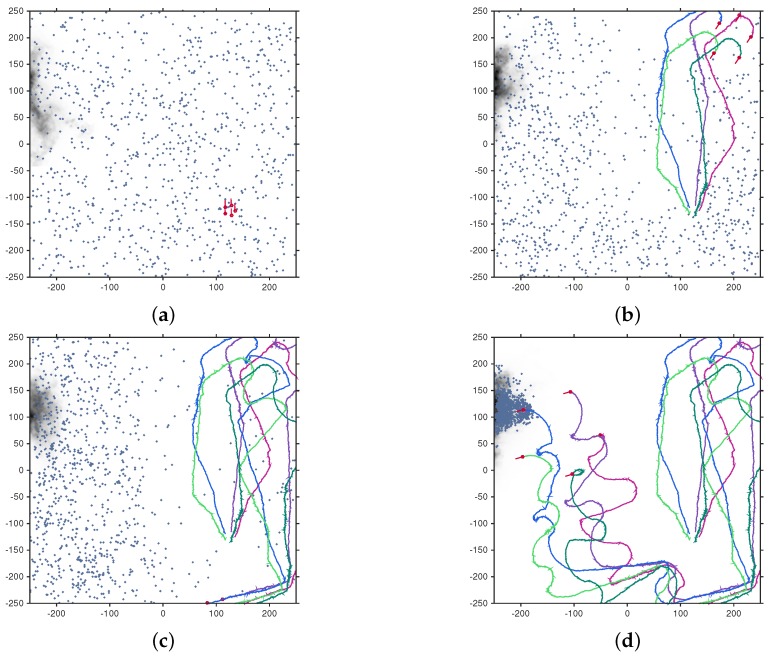
An illustrative run of the multi-robot search algorithm on the experimental dataset using N=5 platforms. (**a**–**d**) show the positions and trajectories of the platforms at times k=0, 10, 20 and 30, respectively. The plume from the experimental dataset can be seen in the top left of the search area, with darker areas representing higher concentrations.

Figure 7 shows an illustrative run of the search algorithm on the experimental dataset at times k=0, 10, 20 and 30. The algorithm terminated, reporting the location of the source, just after the last frame shown, at k=32.

**Figure 8:**
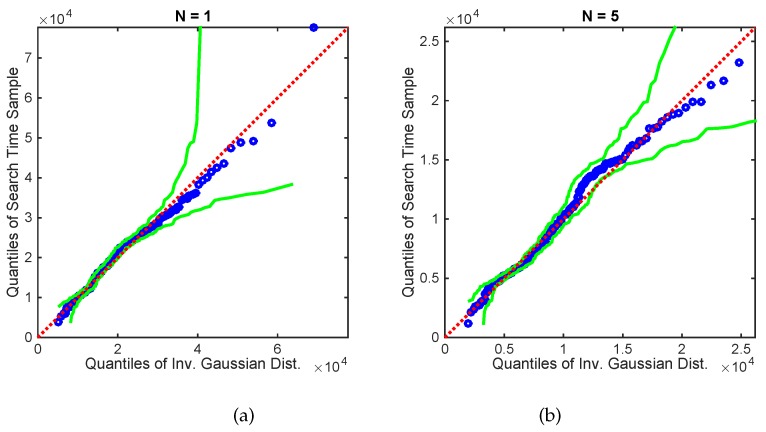
Experimental data: Q-Q plot of the search times for (**a**) N=1 and (**b**) N=5 versus the inverse Gaussian distribution with parameters fitted using maximum likelihood estimation. The source was placed as shown in Figure 7 and initial centroid position was fixed at [125,−125]. The green lines show 95% confidence bands [35].

Figure 8 shows a Q-Q plot of 200 samples of the search times for N=1 and N=5 search platforms versus a fitted inverse Gaussian distribution, with the initial formation centroid position fixed at [125,−125]. As found for the simulated plume in Figure 6, and in our previous work [23], the search times for the experimental plume can be accurately modelled by an inverse Gaussian distribution.

## 6. Summary

The paper proposed an algorithm for autonomous search for an emitting source of hazardous material transported through the environment by a turbulent flow. The search was designed for a group of robots connected in a network with all-to-all communication.

The source parameter estimation was carried out in the Bayesian framework: the posterior density of source strength, conditioned on the source location, was carried out analytically. The posterior density of source location, on the other hand, was computed numerically, using a Monte Carlo method. The source parameter estimation, carried out in this manner, overcomes the problems encountered in previous implementations, such as the assumption that the source strength is known in using the grid-based estimation, or the depletion of particles, when the particle filter is applied.

The robots are moving in a controllable formation, with control parameters including the linear and angular velocity, the travel time and the scale of formation. The reward function for choosing the robot formation control vector was selected as the entropy reduction, with the built-in travel cost.

Numerical results, using both simulated and real data, demonstrate reliable performance: the success rate in finding the source is 100%, with localisation accuracy determined by the termination criterion. Furthermore, the analysis of the algorithm reveals: (1) a diminishing return on increasing the number of platforms in formation; (2) a linear growth of the mean search time with the search area; (3) an increase in the cost of travel resulting in shorter formation displacements; (4) the search displacements are in accordance with the intermittent search strategy; and (5) the PDF of search time for a single searcher is well-modelled by the inverse Gaussian distribution.

There are many avenues for future work. For example, one could explore distributed, rather than centralised, source parameter estimation and robot control. This could take into account a more realistic communication network with limited bandwidth and time delays. The implicit assumption in the presented work was that the search area is an open field. The search in an area with obstacles and known map would be another complementary future research direction: it would require a different dispersion model, and modifications of the parameter estimation and robot control algorithms. Finally, the search in an area with obstacles and an unknown map would require robots to be equipped with appropriate ranging sensors for localisation and mapping. Carrying out autonomously and simultaneously three functions: search, localisation and mapping, is the ultimate goal of this research.

## Supplementary Materials

Supplementary File 1

## Abbreviations

The following abbreviations are used in this manuscript:

*i*	robotic platform index
$\theta_{k}^{i}$	*i*th robot pose at discrete-time *k*
η	parameter vector of emitting source
$Q_{0}$	emitting source strength (release rate)
$r_{0}$	emitting source location, ${\left(X_{0},Y_{0}\right)}^{⊺}$
$z_{1:k}$	the complete measurement vector
$r_{1:k}$	platform/sensor locations corresponding to $z_{1:k}$
$p(\eta |z_{1:k})$	posterior density of η at discrete-time *k*
$u_{k}$	the control vector at time *k*
$\kappa_{k},\vartheta_{k}$	parameters of Gamma distribution at *k*
α	travel cost per unit distance
*U*	average windspeed
*D*	diffusivity
τ	average particle lifetime
*N*	number of robotic platforms
*M*	number of Monte Carlo samples

## Appendix A. Mathematical Derivations

In proving Equations (10) and (11), we will use two properties of gamma distribution:

1. If random variable X∼G(υ,φ), then for any constant c>0, the random variable cX∼G(υ,cφ).
2. Suppose the prior distribution of random variable *Y* is G(υ,φ). Let *n* be a sample from the Poisson distributed likelihood function with parameter *Y*. Then, the posterior distribution of *Y* is $G(\upsilon +n,\frac{\varphi }{1+\varphi })$.

Let us consider first only the concentration measurement taken by a one platform, i.e., N=1, at time k=1, that is a single measurement $z_{1}^{1}$. This measurement was collected at the position $r_{1}^{1}$. The likelihood function of this measurement is $\ell \left(z_{1}^{1}|\eta \right)=P\left(z_{1}^{1};Q_{0}\rho_{r_{0}}\left(r_{1}^{1}\right)\right)$.

Recall that the prior distribution of $Q_{0}$ is $G(\kappa_{0},\vartheta_{0})$. Using property 1 of gamma distribution, the prior density of random variable $Q_{0}\rho_{r_{0}}\left(r_{1}^{1}\right)$ is $G(\kappa_{0},\rho_{r_{0}}\left(r_{1}^{1}\right)\vartheta_{0})$. Using property 2 of gamma distribution, after collecting measurement $z_{1}^{1}$, the posterior distribution of random variable $Q_{0}\rho_{r_{0}}\left(r_{1}^{1}\right)|r_{0},z_{1}^{1}$ can be expressed as $G\left(\kappa_{0}+z_{1}^{1},\frac{\rho_{r_{0}}\left(r_{1}^{1}\right)\vartheta_{0}}{1+\rho_{r_{0}}\left(r_{1}^{1}\right)\vartheta_{0}}\right)$. Finally, using again property 1, the posterior distribution of $Q_{0}|r_{0},z_{1}^{1}$ is given by $G\left(\kappa_{0}+z_{1}^{1},\frac{\vartheta_{0}}{1+\rho_{r_{0}}\left(r_{1}^{1}\right)\vartheta_{0}}\right)$. This proves (10) and (11) for N=1 and k=1. By repeating the sequence of update steps using multiple platforms N>1 over time j=1,2,⋯,k, it can be shown that (10) and (11) holds for any *N* and any *k*.

Next we prove (14). Note that according to Bayes rule, the posterior distribution of signal strength is given by:

(A1) $$p\left(Q_{0}|z_{1:k},r_{0}\right)=\frac{\ell \left(z_{1:k}|Q_{0},r_{0}\right)\pi \left(Q_{0}\right)}{g(z_{1:k}|r_{0})}.$$

Then we have:

(A2) $$\begin{array}{ccc} g(z_{1:k}|r_{0}) & = & \frac{\ell \left(z_{1:k}|Q_{0},r_{0}\right)\pi \left(Q_{0}\right)}{p(Q_{0}|z_{1:k},r_{0})} \end{array}$$

(A3) $$\begin{array}{ccc} & \;= & \frac{G\left(Q_{0};\kappa_{0},\vartheta_{0}\right)\prod_{i=1}^{N}\prod_{j=1}^{k}P\left(z_{j}^{i};Q_{0}\rho_{r_{0}}\left(r_{j}^{i}\right)\right)}{G(Q_{0};\kappa_{k},\vartheta_{k})} \end{array}$$

where $\kappa_{k}$ and $\vartheta_{k}$ are given by (10) and (11), respectively. Upon the substitution of expressions for Gamma and Poisson distributions in (A3), one can show that $Q_{0}$ term cancels out, leading to Equation (14).
